# Activation of posterior ventral pallidum by bitter taste in mice

**DOI:** 10.17912/micropub.biology.001916

**Published:** 2026-01-12

**Authors:** Daisuke H. Tanaka, Tsutomu Tanabe

**Affiliations:** 1 Department of Pharmacology and Neurobiology, Tokyo Medical and Dental University (TMDU)

## Abstract

The posterior ventral pallidum (pVP) is involved in positive affective behaviors and is called the “hedonic hotspot”. However, the responses of the pVP neurons to affective taste stimuli are poorly understood. We examined the expression of *c-fos* , a molecular marker of neuronal activation, in the pVP and surrounding regions after several taste stimuli. The *c-fos* -positive cells were significantly increased in the pVP by bitter taste stimuli, which induced disgust reactions, suggesting that a subset of pVP neurons was activated by bitter taste stimuli associated with negative affective behaviors.

**Figure 1. c-fos expression in pVP after taste stimuli in mice:**
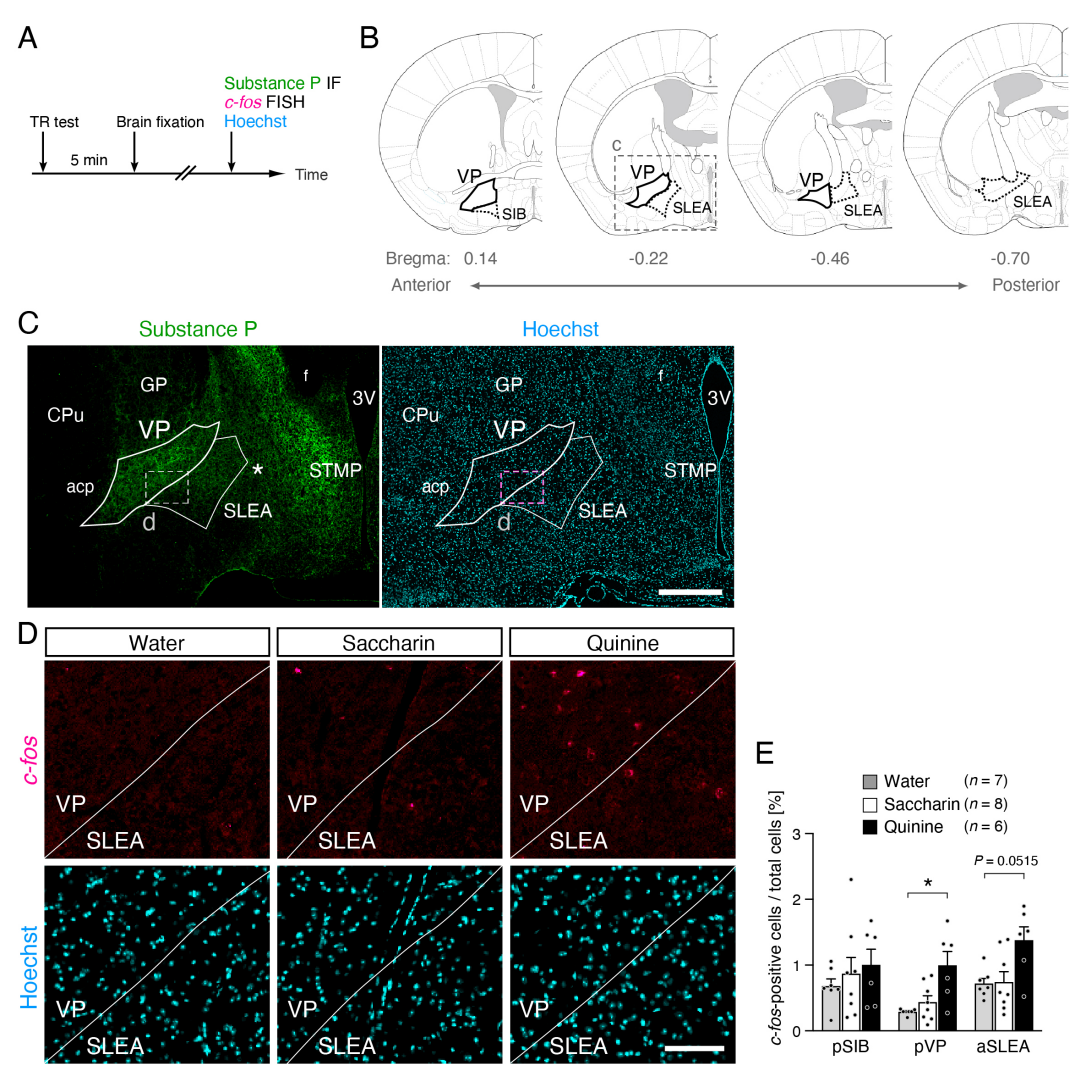
**(A)** Schematic representation of the experimental design for *c-fos* expression analyses after the taste reactivity test. Brains were fixed 5 min after the taste reactivity (TR) test (Tanaka et al., 2019), and brain cryosections were stained for substance P by immunofluorescence (IF), *c-fos* by fluorescence *in situ* hybridization (FISH), and nuclei by Hoechst33258. **(B)** Schematic drawing of the area of the VP (black line), SIB (dotted line), and SLEA (dotted line) in coronal sections analyzed (modified from Franklin and Paxinos, 2008). The numbers at the bottom of each coronal drawing indicate the distance from the bregma (in mm). **(C)** Representative immunoreactivities of substance P (left) and staining by Hoechst33258 (right) in the ventral telencephalon of coronal section at the level of Bregma -0.22 mm (boxed area “c” in **B** ), helping to determine the VP region (white line) (Zahm, 1989; Zahm et al., 1996; Root et al., 2013) with reference to mouse brain atlas (Franklin and Paxinos, 2008). Although substance P immunoreactivity was high in the STMP and VP, the STEP was anatomically far medial to the presumptive VP region, and substance P immunoreactivity was relatively low in the region just lateral to the STEP (asterisk), making it possible to discriminate high substance P immunoreactivity between these regions. Scale bar: 500 µm. **(D)** *c-fos* mRNA expression revealed by fluorescence *in situ* hybridization (upper panels) and Hoechst33258 staining (lower panels) around the border between the VP and SLEA (white line) in coronal section at the level of Bregma -0.22 mm (boxed area with dotted magenta line “d” in **C** ) from mice after intraoral infusion of water, saccharin or quinine solutions. Scale bar: 100 µm. **(E)** The ratio of *c-fos* -positive cells to total cells was significantly higher in pVP with quinine than that with both water and saccharin. The percentage of *c-fos* -positive cells in total cells (Hoechst33258-positive cells) in the posterior SIB (pSIB), posterior VP (pVP), and anterior SLEA (aSLEA) in the coronal sections from mice after intraoral infusion of water ( *n* = 7 mice from 3 independent experiments), saccharin ( *n* = 8 mice from 4 independent experiments), or quinine solution ( *n* = 6 mice from 4 independent experiments) (mean + SEM). Each dot represents the data from each mouse. All the mice used in the taste reactivity test (Tanaka et al., 2019) were analyzed. * *p* < 0.05 (Tukey’s test following two-way RM ANOVA). IF, immunofluorescence; FISH, fluorescence *in situ* hybridization; VP, ventral pallidum; SIB, basal part of the substantial innominata; SLEA, sublenticular extended amygdala; CPu, caudate putamen (striatum); GP, globus pallidus; f, fornix; 3V, third ventricle; STMP, bed nucleus of the stria terminalis medial division, posterior par.

## Description

Positive and negative emotions can be estimated by behavioral liking and disgust reactions, respectively, in the taste reactivity test (Berridge, 2000). In the taste reactivity test, intraoral sweet saccharin stimulation induces liking reactions, such as tongue protrusions, while bitter quinine stimulation induces disgust reactions, such as forelimb flails. The posterior ventral pallidum (pVP) is one of the most crucial regions for liking reactions and is thus called the “hedonic hotspot” (Berridge and Kringelbach, 2015). The neural firing rate in the pVP correlates with the magnitude of liking reactions (Tindell et al., 2006). Pharmacological activation of pVP neurons enhances liking reactions (Smith and Berridge, 2005; Ho and Berridge, 2014). Finally, lesion or pharmacological inactivation of pVP neurons leads to the abolishment of liking reactions, concomitant with enhanced disgust reactions (Cromwell and Berridge, 1993; Shimura et al., 2006; Ho and Berridge, 2014). Given that the VP consists of neurochemically and anatomically heterogeneous populations of neurons (Young et al., 1984; Zahm et al., 1996; Zahm, 1989; Goenewegen et al., 1993; Haber et al., 1993; Churchill and Kalivas, 1994; Bell et al., 1995; Root et al., 2015), the identification and specific labeling of neurons involved in liking and/or disgust reactions at the single-cell level is essential for determining the neural circuits underlying these reactions. One promising strategy for gaining access to neurons involved in specific information processing at the single-cell level is to use immediate early genes (Tonegawa et al., 2015), molecules connecting gene expression to the electrical activity of neurons (Sheng and Greeberg, 1990). However, the expression of immediate early genes, including c-fos, accompanied by liking or disgust reactions, has not been well studied in the pVP. Therefore, investigation of c-fos expression in the pVP accompanied by taste reactivity would be an important first step in identifying neurons underlying taste reactivity and emotions.

​Previously, we found that intraoral saccharin and quinine stimuli significantly induced liking and disgust reactions, respectively, in taste reactivity tests in mice (Tanaka et al., 2019). In the present study, we examined c-fos mRNA expression in the pVP and surrounding regions of these mice 5 min after the taste reactivity test ( Fig. 1A ). Substance P immunoreactivity was procedurally sufficient to determine the VP region with the support of the mouse brain atlas (Franklin and Paxinos, 2008) ( Fig. 1B, C), in agreement with previous studies in rats (Zahm, 1989; Zahm et al., 1996; Root et al., 2013). We found that the ratio of c-fos-positive cells to total cells was not significantly different in any of the brain regions analyzed, including the pVP, between saccharin-infused mice ( Fig. 1D, E, white bars) (n = 8) and water-infused mice ( Fig. 1D, E, gray bars) (n = 7). In contrast, the ratio of c-fos-positive cells to total cells was significantly higher in the pVP of quinine-infused mice ( Fig. 1D, E, black bars) (n = 544 c-fos-positive cells, 46161 Hoechst-positive cells, 77 hemisphere-sections, 6 mice) compared to water-infused mice (P < 0.05, Tukey’s test) (n = 134 c-fos-positive cells, 46158 Hoechst-positive cells, 80 hemisphere-sections, 7 mice). No significant differences were found in the surrounding brain regions, such as the posterior region of the basal part of ​ the substantia innominate (SIB) and the anterior sublenticular extended amygdala (SLEA) ( Fig. 1E ).

We provided evidence that bitter taste stimuli activated some populations of pVP neurons ( Fig. 1D, E), which induces disgust reactions (Tanaka et al., 2019). However, we did not find any significant increase in the number of c-fos-positive cells in the pVP in saccharin-infused mice compared to that in water-infused control mice ( Fig. 1D, E) in contrast to previous findings, the firing rate of neurons in the pVP was found to increase significantly by sucrose infusion, which induced liking reactions compared to the water control or high-concentration salt solution that induced disgust reactions (Tindell et al., 2006). A possible explanation for this discrepancy may be the difference in the duration of neural activity monitoring in each study. While Tindell et al. (2006) recorded neural activity for only 1 s after intraoral infusion, we detected c-fos expression 5 min after the completion of the taste reactivity test, in which intraoral infusion lasted for 2 min in total. c-fos expression in the present study reflects total neural activity during the taste reactivity test. Thus, one of the coherent hypotheses would be that the pVP neurons dramatically increase their firing rate for only a few seconds and subsequently become quiet when sweet taste stimuli are provided. On the other hand, the pVP neurons may moderately increase their firing rate for a longer period due to the bitter taste stimuli, and the moderate but longer firings may induce more c-fos expression than strong but short firings. Additional electrophysiological or optical recordings of pVP neurons for a longer period during intraoral infusion are needed to test this hypothesis.

In conclusion, although pVP is thought to be a hedonic hotspot, a subset of neurons is activated by bitter taste stimuli associated with negative affective behaviors.

## Methods

The present study used brain samples obtained from a previous study (Tanaka et al., 2019). For details on the animals used, surgery, behavioral analysis, and brain fixation, please refer to Tanaka et al. (2019).

***Fluorescence in situ hybridization combined with Immunofluorescence***

Fluorescence *in situ* hybridization combined with immunofluorescence was performed as previously described (Tanaka et al., 2019). Frozen coronal sections (10 µm) were incubated in Proteinase K solution (1 µg/mL), washed with PBS, and incubated in acetylation buffer (90.6 mM triethanolamine, 0.216N HCl, 21.2 mM acetic anhydride). After washing in PBS with 0.3% Triton X-100 and PBS, the sections were incubated in hybridization solution (5x SSC, 50% Formamide, 5x Denhardt’s solution, 250 µg/mL yeast total RNA) and then with digoxigenin-labeled *c-fos* riboprobes (Bepari et al. 2012) in the hybridization solution overnight at 65 °C. After washing with 0.2 × SSC (3.33 mM trisodium citrate dihydrate, 3.33 mM sodium chloride), the sections were incubated with blocking solution and then with rabbit anti-substance P antibody (1:500; code#20064; Immunostar). After washing, the sections were incubated with alkaline phosphatase-conjugated sheep anti-digoxigenin antibody (1:2000; code#11093274910; Roche) and Alexa488-conjugated donkey anti-rabbit IgG antibody (1:800; Molecular Probes). After washing, sections were incubated with HNPP/Fast Red TR solution (code#11758888001; Roche), washed, incubated with Hoechst33258 (1/1000) in PBS for nuclear staining, and mounted with CC/Mount (code#K002; Diagnostic Biosystems). Images were captured using a x10, 0.45 numerical aperture objective with a BZ-9000 microscope (Keyence). The sequence specificity of *the c-fos* riboprobes used in the present study has been confirmed in previous studies (Bepari et al. 2012).

***Quantification of c-fos-positive cells***

We counted the number of *c-fos* -positive cells manually in the following brain regions and coronal sections: posterior region of SIB in sections ranging from bregma 0.14 mm to -0.1 mm, pVP in sections ranging from bregma 0.14 mm to -0.46 mm, which largely corresponds to so called “hedonic hotspot” in the pVP in rats (Cromwell and Berridge, 1993; Smith et al., 2005; Ho and Berridge, 2014), anterior SLEA in sections ranging from bregma -0.22 mm to -0.7 mm, which appears to correspond to a part of hedonic hotspot in the posterior VP/SI regions in rats (Cromwell and Berridge, 1993). The total number of cells in each brain region was examined by counting the number of cell nuclei stained with Hoechst33258 by using NIH ImageJ software (version 1.40 g). Brain regions in coronal sections were determined based on fluorescent images of nuclear staining by Hoechst33258 and substance P staining, which labels the entire VP (Zahm, 1989; Zahm et al., 1996; Root et al., 2013), with reference to the mouse brain atlas (Franklin and Paxinos, 2008).

***Statistical analysis***

The numbers of *c-fos* -positive cells and total cells in each brain region from each animal were summed, and then the ratio of *c-fos* -positive cells to total cells in the specific brain region from the specific animal was calculated. All data are expressed as the mean ± SEM. All data were analyzed using two-way repeated measures ANOVA followed by Tukey’s multiple comparison test. Statistical significance was set at P < 0.05. All tests were performed using the two-tailed method
